# β-Cyclodextrin–Hyaluronic Acid Polymer Functionalized Magnetic Graphene Oxide Nanocomposites for Targeted Photo-Chemotherapy of Tumor Cells

**DOI:** 10.3390/polym11010133

**Published:** 2019-01-14

**Authors:** Wenting Liang, Yu Huang, Dongtao Lu, Xuewen Ma, Tao Gong, Xiaodong Cui, Baofeng Yu, Cheng Yang, Chuan Dong, Shaomin Shuang

**Affiliations:** 1 Institute of Environmental Science, Department of Chemistry, Shanxi University, Taiyuan 030006, China; huangyu920323@163.com (Y.H.); ludongtao@sxu.edu.cn (D.L.); maxuewen@sxu.edu.cn (X.M.); cuixiaodong@sxu.edu.cn (X.C.); dc@sxu.edu.cn (C.D.); 2 Department of Biochemistry and Molecular Biology, Shanxi Medical University, Taiyuan 030001, China; gyt830626@163.com; 3 State Key Laboratory of Biotherapy, College of Chemistry, Sichuan University, 29 Wangjiang Road, Chengdu 610064, China

**Keywords:** β-cyclodextrin, hyaluronic acid, magnetic graphene oxide, near-infrared, photo-chemotherapy

## Abstract

A multifunctional targeted drug delivery platform (CDHA–MGO) has been successfully constructed by grafting β-cyclodextrin–hyaluronic acid polymers (CDHA) to Fe_3_O_4_–graphene oxide (MGO). The obtained CDHA–MGO nanocomposite has good water-dispersibility, easy magnetic separation, high near-infrared (NIR) photothermal heating, and excellent biocompatibility. The β-cyclodextrin-hyaluronic acid polymers efficaciously enhance the doxorubicin (DOX) loading amount up to 485.43 mg·g^−1^. Meanwhile, the Fe_3_O_4_–graphene oxide provides a facile photothermal response mechanism to handle the NIR-triggered release of DOX in weak acidic solvent environments. Significantly, the DOX-loaded nanocomposite (DOX@CDHA–MGO) has displayed CD44 receptor-mediated active targeting recognition and chemo-photothermal synergistic therapy of hepatoma cells. These findings suggest that the as-prepared drug delivery platform would be of valuable potential for cancer-targeted photo-chemotherapy.

## 1. Introduction

As one kind of fatal disease, cancer causes over eight million deaths every year in the world. For most cancer conditions, chemotherapy is regarded to be the treatment of cancer in a standardized regimen [1,2,3]. The decades of researches in chemotherapy demonstrate that the major anti-cancer drugs are confronted with premature degradation, injury to healthy cells, and insufficient localization to tumor sites [4,5]. To avoid these obstacles and risks of chemotherapy, scientists are increasingly paying great attention to some new therapeutic strategies for cancer management [6,7,8,9]. Photo-chemotherapy (PCT) is an increasingly recognized alternative to treat various cancers in previous reports [10,11,12]. The PCT system utilizes near-infrared (NIR) light irradiation to promote the apoptosis of cancer cells by heating and trigger the release of drug from the carrier, and exhibits many good features such as being minimally invasive, controllable, and highly efficient [13,14]. Various functional nanomaterials, such as carbon nanomaterials [15], magnetic nanoparticles [16,17], gold nanoparticles [18], and semiconductor quantum dots [10] have been investigated to develop PCT therapeutic approaches.

Graphene oxide (GO), as a two-dimensional (2D) carbon nanomaterial, has received fascinating research attention in drug delivery systems. GO possesses unique features, such as facile synthesis, large specific surface area, abundant functional groups, good biocompatibility, and near-infrared (NIR) absorbance, which create an opportunity to prepare drug carriers for cancer photo-chemotherapy (PCT) [15,19]. Recently, a widely prescribed strategy to achieve efficient external targeting is to integrate GO with magnetic nanoparticles to form a hybrid, which can drive the drug carriers by an external magnetic field both in separation and tumor treatment. Fe_3_O_4_ nanoparticles have a great deal of advantages over other magnetic nanoparticles, such as strong superparamagnetism, low toxicity, excellent biocompatibility, easy preparation process, and near-infrared (NIR) absorbance, and thus have been widely used to construct magnetic graphene oxide (MGO) drug carriers [20,21,22]. However, their therapeutic utilization is mainly limited by their easy agglomeration and decreasing magnetic properties with oxidization. Otherwise, with the development of research, dual-targeting drug delivery systems have been attracted much attention based on the immobilization of targeting molecules on magnetic nanomaterials.

In this context, polymer modification is a promising approach for improving targeting and enabling high delivered doses, while minimizing the toxic side effects to healthy tissue. As a biocompatible polysaccharide, hyaluronic acid (HA) is one favorable candidate, with a targeting function which can bind to Cluster of Differentiation 44 (CD44) receptors over-expressed on the surface of cancer cells with high affinity [23,24,25]. To further enhance the drug loading capacity, it has been attempted to link β-cyclodextrin (β-CD) onto the polymeric network to give enhanced bioavailability, solubility, and stability to the drug molecules, due to its cylindrical shape with a hydrophilic exterior and lipophilic interior cavity [26]. Recently, β-cyclodextrin-grafted hyaluronic acid polymers have been utilized and developed to form a biocompatible nanocarrier, which coalesced the advantages of both β-CD’s cavity, to include hydrophobic molecules, and HA’s polymeric skeleton, to selectively recognize CD44 for targeting [27,28,29,30]. These findings triggered our research interests to design a β-CDHA-modified nanocarrier as a multifunctional intelligent photothermal therapy platform.

**Scheme 1:**
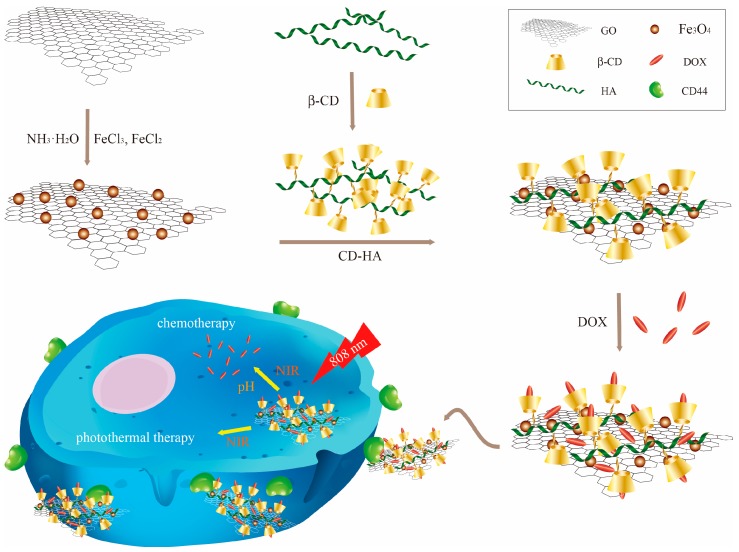
Illustration of the synthesis of β-cyclodextrin–hyaluronic acid polymers grafted with Fe_3_O_4_–graphene oxide (CDHA–MGO) for targeted photo-chemotherapy of tumor cells.

Herein, we report the novel design of a photo-chemotherapy nanocarrier CDHA–MGO by the conjugation of biocompatible and hydrophilic β-Cyclodextrin–hyaluronic acid (CDHA) polymers with Fe_3_O_4_ magnetic nanoparticle-grafted GO nanosheets (MGO), as shown in Scheme 1. The CDHA polymers not only enhanced the stability of MGO in physiological conditions, but also offered the capability of targeting cancer cells with HA receptors. Afterwards, a common anti-cancer drug, doxorubicin (DOX), was loaded into the CDHA–MGO by host–guest interaction and hydrophobic interaction. The DOX-loaded nanocomposite (DOX@CDHA–MGO) exhibited NIR-triggered sustainable releases in the acid environment of tumors. Moreover, the cell uptake experiment of DOX@CDHA–MGO in vitro displayed CD44 receptor-mediated active targeting recognition and chemo-photothermal synergistic therapy of hepatoma cells.

## 2. Materials and Methods

### 2.1. Materials

Graphite powder, 1,2-ethanediamine (EDA), dimethylformamide (DMF), and hyaluronic acid (HA) were purchased from Macklin (Shanghai, China). Iron chloride tetrahydrate (FeCl_2_·4H_2_O) and ferric chloride hexahydrate (FeCl_3_·6H_2_O) were bought from the Guangfu Fine Chemical Industry Research Institute (Tianjin, China). β-CD, P-toluenesulfonyl chloride (p-TsCl), 3-aminopropyltriethoxysilane (APTES), *N*-hydroxysuccinimide (NHS), and 1-ethyl-3(3-dimethylaminopropyl) carbodiimide (EDC) were supplied by Aladdin (Shanghai, China). Doxorubicin hydrochloride (DOX) was purchased from Civi Chemical Technology Co. Ltd. (Shanghai, China). 3-(4,5-dimethyl-2-thiazolyl)-2,5-diphenyl-2*H*-tetrazoliumbromide (MTT) was purchased from Sigma-Aldrich (St. Louis, MO, USA). The human hepatoma cell line BEL-7402 was purchased from the cell bank of the Shanghai Institute for Biological Sciences (Shanghai, China). All the other chemicals were obtained in analytical grades from local suppliers and used without further purification. Deionized water (DI water) was prepared by a Milli-Q Plus system (Millipore, Burlington, MA, USA) and used throughout this experiment.

### 2.2. Preparation of CDHA-MGO

#### 2.2.1. Synthesis of MGO 

The GO nanosheet was prepared by a modified Hummer’s method with natural graphite powder. The graphite powder (3.0 g, 1 wt equiv) was oxidized with strong oxidants, including KMnO_4_ (18.0 g, 6 wt equiv) and a 9:1 mixture of concentrated H_2_SO_4_/H_3_PO_4_ (360:40 mL), under vigorous stirring for 12 h at 50 °C. The reaction was cooled to room temperature and further reacted with 30% H_2_O_2_ (3 mL). The resulting mixture was centrifuged (4000 rpm for 30 min), and the solid was washed with 10% hydrochloric acid, ethanol, and DI water several times repeatedly, until the pH of the supernatant was neutral. After further centrifuging, the resulting solid was freeze-dried for 24 h to get the GO nanosheet [31]. FeCl_3_·6H_2_O and FeCl_2_·4H_2_O (50 mg at a M ratio of 2:1) were used as Fe sources, and were dissolved into 100 mL DI water with dispersed GO nanosheets (200 mg). Then, NH_3_·H_2_O (25%) was quickly injected into the solution. The reaction was kept stirred with vigorous mechanical agitation under a nitrogen environment for 3 h at 80 °C to yield a magnetic black precipitate. The mixture was magnetically separated, and washed with DI water and anhydrous ethanol repeatedly. Finally, the prepared products were named as MGO, and were freeze-dried overnight for further use. 

The obtained MGO powder (200 mg) was ultrasonically dispersed in ethanol (200 mL, 99.5%) for thirty minutes to ensure that it was completely dispersed. Then APTES (300 μL) was added to the mixture with mechanical agitation for 8 h at room temperature under N_2_ protection. The precipitate was washed with ethanol and DI water three times, and then collected by magnetic separation. Next, it was freeze-dried for 24 h to obtain amino-MGO.

#### 2.2.2. Synthesis of CDHA-MGO

**Scheme 2:**
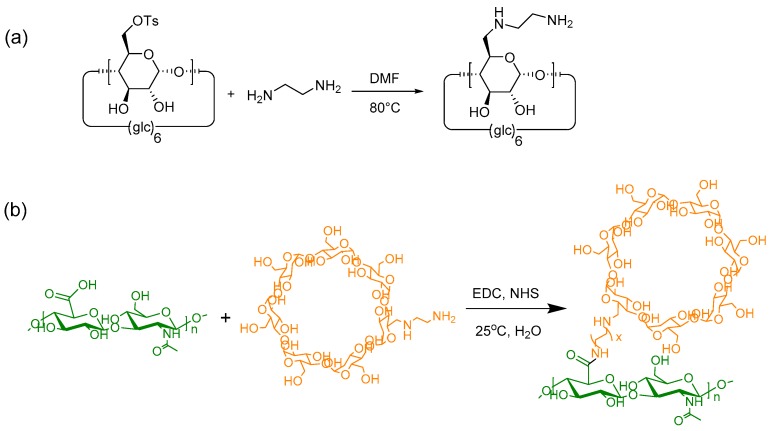
Synthetic scheme of mono-6-deoxyl-6-ethylenediamino-β-cyclodextrin (**a**) and β-cyclodextrin–hyaluronic acid (CDHA) polymer (**b**).

The CDHA polymer was synthesized by means of an amide condensation reaction between mono-6-deoxyl-6-ethylenediamino-β-CD and HA sodium salt with EDC–NHS, according to a reported method [27], as shown in Scheme 2. Mono-6-deoxy-6-(p-tolylsulfonyl)-β-cyclodextrin (M-6-O-Ts-β-CD, 5 g, 3.9 mmol, 1 equiv) and an excess amount of EDA (10.55 g, 175.5 mmol, 45 equiv) were dissolved in DMF (25 mL) and reacted with magnetic stirring under nitrogen at 80 °C for 18 h. After the mixture was cooled to room temperature, the product was precipitated from excess cold acetone three times to remove the residue EDA, and dried under vacuum to afford the final product as a white powder (β-CD–EDA). The chemical structures of β-CD–EDA were confirmed by ^1^H and ^13^C NMR spectroscopy (Figure S1). Then, β-CD–EDA was conjugated with HA using the standard EDC–NHS coupling reaction. In brief, HA (500 mg, 1.24 mmol) dispersion in DI water (100 mL) was activated by an EDC–NHS (2.48 mmol) solution, and the solution was treated by continuous stirring for 30 min at 25 °C. β-CD–EDA (200 mg, 0.17 mmol) was added into the flask above, and the mixed solution was allowed to react at room temperature for 24 h. The resulting solution was dialyzed in DI water over 5 days to purify, and freeze-dried to get the CDHA polymer as a white powder. The obtained white powder was characterized by ^1^H NMR spectroscopy (Figure S2). 

Moderate EDC–NHS (0.5 mmol) was added into the CDHA aqueous solution (100 mg, 20 mL) with continuous stirring for 2 h at 25 °C. Then, amino-MGO (100 mg) was added into the above mixture and the reaction was stirred at room temperature for 48 h. The obtained product was collected by the aid of an external magnet and washed with ethanol and DI water three times. Finally, CDHA–MGO was obtained after lyophilization.

### 2.3. Characterizations of CDHA–MGO

Fourier transform infrared (FT-IR) spectroscopy measurements were recorded on a Bruker Tensor 27 (Ettlingen, Germany), using KBr as the background in the 4000–400 cm^−1^ range. Transmission electron microscopy (TEM) was performed on a FEI Tecnai G2 F30 (Jeol, Tokyo, Japan), which was also equipped with energy dispersive X-ray spectrometer (EDX) analysis. X-ray diffraction (XRD) was carried out on a Bruker D8 Advance diffractometer in the 2θ range of 10–80°. For thermogravimetric analysis (TGA) measurements, the weight loss of the dried sample was monitored on a TA Instruments Q50 (TA, New Castle, DE, USA), under a N_2_ flow at a rate of 10 °C·min^−1^. Magnetic properties were measured with a Quantum Design Verslab vibration sample magnetometer (VSM, Quantum Design, San Diego, CA, USA) at 300 K. Zeta potentials and dynamic light scattering (DLS) were measured at 25 °C using a Zetasizer Nano-Zs (Malvern Instruments, Malvern, UK). Nitrogen adsorption–desorption measurements were carried out on a Micromeritics ASAP 2020 instrument. UV-Vis-NIR spectra measurements were carried out using a Lambda-950 spectrometer (PerkinElmer, Waltham, MA, USA). Fluorescence (FL) measurements were performed on a FP-8300 fluorescence spectrophotometer (JASCO, Tokyo, Japan). Fluorescence images were observed using an Olympus IX70 inverted fluorescent contrast phase microscope (Olympus, Tokyo, Japan). ^1^H and ^13^C NMR spectra were measured on a Bruker instrument (AVANCE III HD-600 MHz) NMR spectrometer.

### 2.4. NIR Enhanced Temperature Measurement

Solutions containing CDHA–MGO with different concentrations (0.2, 0.4, 0.8, and 1.2 mg·mL^−1^) were exposed to an 808 nm laser with different power densities for 10 min. Phosphate-buffered saline (PBS) (pH 5.3), GO, and MGO (0.4 mg/mL) were selected as negative control experiments in our design. The enhanced temperatures of various samples were collected at 2 min intervals. 

### 2.5. Drug Loading and Release Behaviors of CDHA–MGO

Doxorubicin hydrochloride (DOX) was used to investigate the drug loading and controlled release performance. The drug loading capacity of CDHA–MGO was determined by adding different amounts of DOX to a fixed concentration of CDHA–MGO. The suspension was shaken in a thermostatic bath shaker at a speed of 200 rpm at room temperature for 6 h in the dark at 25 °C. After equilibrium was reached, the solid sample was separated using a permanent magnet, and the supernatant was collected to determine the drug loading content using a FL spectrophotometer at 558 nm (Figure S3). The DOX loading capacity of CDHA–MGO was calculated according to the following formula (Equation (1)):

*Q_e_* = (*M*_0_ − *M*_e_)/*M_n_* (1)

where *M*_0_ and *M_e_* are the total mass of DOX dissolved in the initial solution and the mass of DOX remaining in the supernatant solution, respectively. *M_n_* is the mass of the CDHA–MGO (mg).

For the drug loading kinetic experiments, 1 mg of CDHA–MGO was added to 4mL PBS (pH 7.4) containing DOX (0.01 mg/mL), and mixed thoroughly. The suspension was shaken in a thermostatic bath shaker at a speed of 200 rpm at 25 °C in the dark. At various time intervals, samples were collected after magnetic separation, and fluorescence intensities were measured to calculate loading capacity.

In order to investigate the pH/temperature-responsive release of DOX, drug-loaded DOX@CDHA–MGO was studied in phosphate-buffered saline (PBS, 0.01 M) at pH 5.3 (the cytoplasm pH of cancer cells) and 7.4 (the physiological pH) at 25, 37, and 40 °C. The investigation of the NIR-responsive release of DOX@CDHA–MGO was carried out in PBS (pH 5.3) with NIR irradiation (808 nm laser) of 2 W·cm^−1^ for 10 min. The release behavior was studied under constant stirring, and quantitative solutions were taken out for fluorescence measurements at a series of time points. The concentration of DOX released from CDHA–MGO was measured by fluorimetry at 558 nm. The percentage of drug released was given as the following equation (Equation (2)):

(2) $$\mathrm{The}\;\mathrm{percentage}\;\mathrm{of}\;\mathrm{drug}\;\mathrm{released}\;=\frac{M_{\left(\mathrm{the}\;\mathrm{released}\;\mathrm{amount}\;\mathrm{of}\;\mathrm{drug}\right)}}{M_{\left(\mathrm{the}\;\mathrm{loading}\;\mathrm{amount}\;\mathrm{of}\;\mathrm{drug}\right)}}\;\times \;100\%$$

### 2.6. Cellular Uptake Assays and Anti-cancer Activity Evaluation

The cellular uptake of DOX@CDHA–MGO was researched by fluorescence microscopy. BEL-7402 cells (a type of human hepatoma cell with abundant HA receptors being overexpressed on its surface) were used for this experiment. The BEL-7402 cells were seeded into 24-well plates. After 24 h incubation, the cells were incubated with DOX@CDHA–MGO (20 μg/mL) at 37 °C. At different time points (0.5 h, 1 h, 2 h, and 4 h), the media was removed from the wells and the cells were carefully washed with phosphate-buffered saline (PBS), followed by observation under an inverted fluorescence microscope.

To study the effect of chemotherapy and photothermal therapy, LIVE/DEAD Kits (a mixture of Hoechst 33342 and propidium iodide (PI) solution) were applied to visualize cell death. CDHA–MGO and DOX@CDHA–MGO (20 μg/mL) were incubated with BEL-7402 cells in 96-well microplates for 4 h. Then, the culture media was replaced with fresh media, and corresponding wells were irradiated for 5 min with an 808 nm NIR laser (2 W/cm^2^) for thermal therapy. After the cells were cultured for a further 8 h, cells were co-stained by LIVE/DEAD Kits for 20 min. Live cells exhibited a blue color, and dead cells showed a red color. Repetitive studies were carried out, and three independent fields were examined in each study.

### 2.7. Cell Viability Assay 

MTT assays were carried out to quantify the cytotoxicity of CDHA–MGO and DOX@CDHA–MGO. BEL-7402 cells were cultured in 96-well plates with 100 μL of media at a density of 5 × 10^3^ cells/well for 12 h at 37 °C. After the attachment of the cells, different concentrations (10, 20, and 40 μg/mL) of CDHA–MGO or DOX@CDHA–MGO were added into the 96-well plates and incubated for 4 h. The culture media was then replaced with fresh media, and corresponding wells were irradiated for 5 min with a NIR laser (808 nm, 2 W/cm^2^) for thermal therapy. After the cells were cultured for a further 20 h, samples were carefully washed and treated with MTT for another 4 h. Then, dimethylsulfoxide (DMSO) was added to these samples to dissolve the formazan crystals. After that, the absorbance was measured with a microplate reader at 492 nm. The half-maximal inhibitory concentration (IC_50_) was calculated by regression analysis of cell viability. 

## 3. Results

### 3.1. Characterization of CDHA–MGO

**Figure 1:**
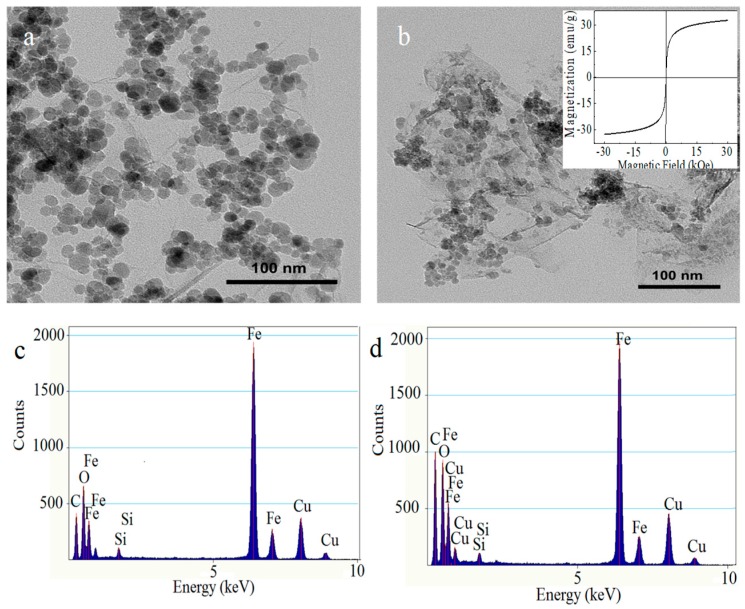
(**a**) TEM images of Fe_3_O_4_–graphene oxide (MGO); (**b**) TEM image of CDHA–MGO (insert: The magnetization curves of CDHA-MGO at 300 K); (**c**) energy dispersive X-ray spectrum (EDX) of MGO; (**d**) EDX of CDHA–MGO.

To demonstrate the morphology and size of the as-prepared nanohybrid, representative TEM was used to characterize the surface morphologies. As shown in Figure 1a, a large amount of uniform Fe_3_O_4_ magnetic nanoparticles (MNPs) are distributed on the surfaces and edges of the GO nanosheet homogeneously, with an average size of about 14 ± 2 nm, and almost no unbonded Fe_3_O_4_ is viewed outside of GO sheets. After the modification of CDHA, it can be clearly observed that the CDHA covers on the surface of MGO like a film, as shown in Figure 1b. The TEM images could provide direct evidence for the successful fabrication of CDHA–MGO. Furthermore, the EDX spectra show the elemental fingerprints of the nanocomposite, as illustrated by Figure 1c,d. The EDX spectra of MGO show strong peaks of Fe, C, and O, which demonstrate the presence of MNPs. Compared with MGO, the higher C and O peaks of CDHA–MGO further clarify the fact that CDHA has been anchored on the surface of MGO successfully.

The magnetic properties of the as-prepared CDHA–MGO were investigated with a VSM at 300 K. As shown in the Figure 1b insert, it is evident that the CDHA–MGO possesses a good magnetic property, with the magnetic saturation value at 32.46 emu·g^−1^, implying that Fe_3_O_4_ has been successfully deposited on the surface of the GO in situ and the magnetic properties were kept well during the functionalized process. Furthermore, the magnetization curve of the sample shows no remnant magnetization or coercivity, which indicates that CDHA–MGO is a superparamagnetic nanomaterial at room temperature and could be used for magnetic separation and targeted drug delivery.

The size information of MGO and CDHA–MGO nanocomposites were also determined by dynamic light scattering (DLS) in aqueous solution, and the hydrodynamic diameter distribution is shown in Figure S3. The average diameter of MGO was 186.9 nm, and the average diameter was 338.5 nm for CDHA–MGO. Comparing with MGO, the significantly larger hydrodynamic diameter of CDHA–MGO is likely attributable to the variation of its hydration layer and shape after conjugation with CDHA polymers.

**Figure 2:**
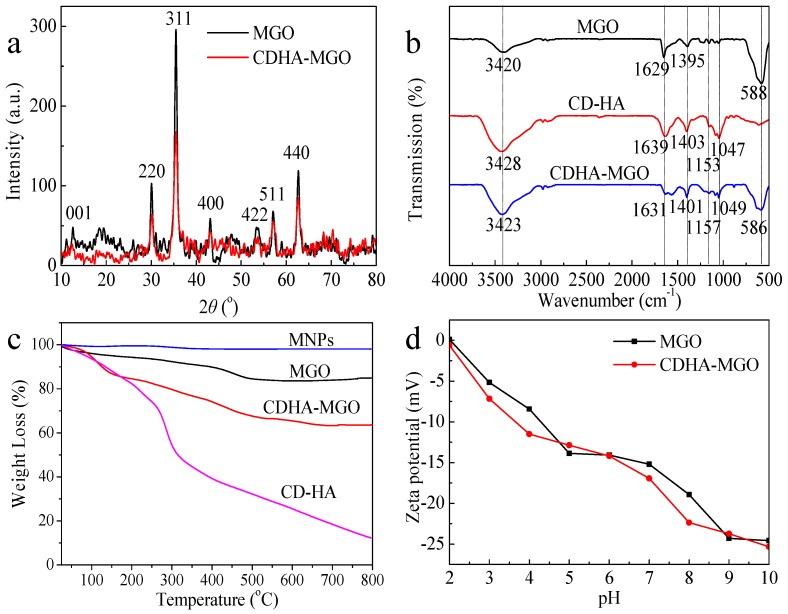
(**a**) XRD patterns of the MGO and CDHA–MGO; (**b**) FTIR spectra of MGO, CDHA and CDHA–MGO; (**c**) TGA curves of magnetic nanoparticles (MNPs), MGO, CDHA, and CDHA–MGO; (**d**) Zeta potentials of MGO and CDHA–MGO at different pH values.

The XRD diffraction patterns of MGO and CDHA–MGO are shown in Figure 2a, which enables examination of the phase purity. The peak at 2θ = 11.02 (001) is a feature diffraction peak of exfoliated GO [20]. Six characteristic peaks are at 2θ = 30.31 (220), 35.58 (311), 43.19 (400), 53.58 (422), 57.18 (511), and 62.87 (440), which respectively correspond to the diffraction planes of iron oxide according to the standard XRD data cards of Fe_3_O_4_ crystals (JCPDS No. 85-1436) [32]. As compared with MGO, the CDHA–MGO nanocomposites display all diffraction peaks of Fe_3_O_4_, and no obvious shifts can be observed, indicating that Fe_3_O_4_ nanoparticles have been successfully connected with GO and have no effect on the structure during the functionalized process. In addition, according to the XRD pattern, the crystal size of the Fe_3_O_4_ on CDHA–MGO can be evaluated to be 14.95 nm using Scherrer’s formula, which is similar to the results of the TEM.

To further characterize the formation of CDHA–MGO together with individual MGO and CDHA, corresponding FTIR **s**pectra were performed in Figure 2b. For the curve of CDHA, the bands at 3400 and 1647 cm^−1^ are ascribed to the stretching vibrations of O–H and C=C, respectively. Significant peaks were observed in the range of 1200–1000 cm^−1^, corresponding to the characteristic peak of CDHA. The FTIR spectrum of MGO reveals the characteristic peak around 584 cm^−1^, corresponding to the shifted stretching vibration of the Fe–O bond compared to that of bare Fe_3_O_4_, illustrating that Fe_3_O_4_ is successfully bound to the GO. After the MGO nanohybrid was further modified with CDHA, it could be observed that the FTIR spectrum of CDHA–MGO exhibits the characteristic absorbing peaks of CDHA. The band at 1157 cm^−1^ is assigned to the coupled C–O–C stretching/O–H bending vibrations. The featured peaks at 1040 and 1079 cm^−1^ are attributed to the coupled C–O/C–C stretching/O–H bending vibrations. The above results demonstrate that CDHA has been anchored on the surface of MGO successfully.

The amount of CDHA grafted onto the MGO was evaluated from TGA analyses. As revealed in Figure 2c, the TGA curve of the bare Fe_3_O_4_ MNPs exhibits almost no weight losses during the full temperature range. For MGO, a slighter weight loss is observed between 200 and 600 °C, which may be due to the removal of the labile oxygen-containing functional groups on MGO. The TGA curve of the CDHA polymers displays a significant weight loss at about 300 °C under the nitrogen flow, which should be attributed to the decomposition of CDHA. In the same condition, the TGA curve of CDHA–MGO showed two steps of mass loss, of which the step below 150 °C is ascribable to the loss of adsorbed water in the sample, and the second major mass reduction from 150 to 700 °C could be attributed to the decomposition of CDHA. The superficial polymers modified on MGO are destroyed firstly, and the internal chemical groups which bind with the MGO are further destroyed as temperatures rise. In contrast to the evident weight loss of pure CDHA polymers at around 300 °C, the decomposition of CDHA polymers conjugated on the surface of MGO is a gradual decomposition process with increasing temperature, which suggests that the thermostability of the polymers were enhanced after binding with the MGO. In addition, the total mass loss of CDHA over the temperature range was estimated to be 23.5%, which was estimated to be 128.8 mg/g.

The zeta potential measurements can define the surface charge of MGO and CDHA–MGO under different pH conditions, and further revealed the stability of as-prepared MGO and CDHA–MGO in aqueous solution. As shown in Figure 2d, the zeta potentials of both MGO and CDHA–MGO are below 0 mV at pH 3–10, and the values of CDHA–MGO were lower than naked MGO. This behavior is attributed to the presence of the highly negatively charged carboxylic groups (–COO) from CDHA, which further indicates that the grafting of CDHA successfully coated onto the MGO. The high absolute zeta potential values prove that CDHA–MGO suspensions have good stability in the solution, according to the colloidal stability criteria defined by ASTM [33].

The results the of N_2_ adsorption–desorption isotherm and pore size distribution curves (Figure S4) indicate that MGO possesses large Brunauer-Emmett-Teller (BET) surface area (329 m^2^/g) and pore volume (0.954 cm^3^/g), and a uniform pore diameter (D) of 8.5 nm. Comparing with MGO, CDHA–MGO exhibits a BET surface area of 228 m^2^/g, a pore volume of 0.425 cm^3^/g, and a uniform pore diameter (D) of 7.4 nm, which is slightly reduced after the CDHA polymers grafted onto the surface of MGO. The large BET surface area and mesoporous structure in the hybrid CDHA–MGO could provide favorable conditions for drug loading and release.

### 3.2. Drug Loading Behavior

**Figure 3:**
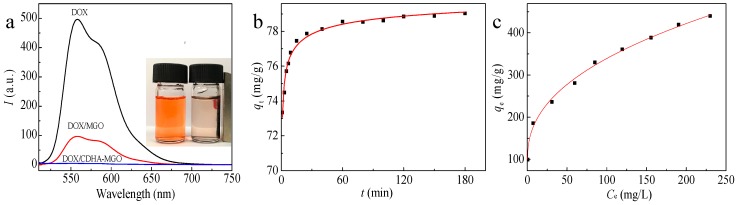
(**a**) The fluorescence spectra of 0.0025 mg/mL doxorubicin (DOX), the supernatant of DOX/MGO, and the supernatant of DOX/CDHA–MGO (inserts display the DOX (left) and DOX/CDHA–MGO by magnetic separation (right)); (**b**) adsorption kinetic curves of CDHA–MGO towards DOX (0.01 mg/mL) at pH 7.4; (**c**) adsorption equilibrium isotherm profiles for DOX on CDHA–MGO.

To assess the drug loading capacity of CDHA–MGO, we selected doxorubicin (DOX) as a model drug to load onto the surface of MGO and CDHA–MGO by a simple mixture and vibrating approach via π–π stacking, hydrophobic interactions, and host–guest interaction between functionalized GO and drugs. As shown in Figure 3a, the fluorescence intensity of DOX is reduced by more than 98% with the CDHA–MGO, while an only 80% decrease is observed for MGO, indicating a higher efficient loading on CDHA–MGO than MGO. The higher drug adsorption efficiency of CDHA–MGO results from the modification of CDHA polymers providing a hydrophobic void and cavity. The insert image demonstrates that the DOX could be effectively adsorbed by CDHA–MGO and attracted quickly toward the magnet with an external magnetic field, giving rise to the solution becoming clear and transparent. In addition, the pH effects on DOX loading of CDHA–MGO were investigated from pH 3.0 to 11.0 at 25 °C, with an initial drug concentration of 0.03 mg mL^−1^. As displayed in Figure S6, it can be observed that the loading capacity of CDHA–MGO for DOX stays high at pH 6.0 to 8.0, but decreases greatly when the pH is in an acidic or basic range. In the acidic or basic environments, the hydrogen bonding and hydrophobic interactions between DOX and CDHA–MGO are significantly weakened by the protonation of DOX, which greatly reduces the loading capacity of CDHA–MGO for DOX [20,34]. Hence, the physiological pH 7.4 was used in the subsequent experiments.

The loading process of DOX onto CDHA–MGO is a dynamic equilibrium process. In order to study the influence of the adsorption time, the loading amounts of CDHA–MGO for DOX in the initial concentration of 0.01 mg/mL under the different times at pH 7.4 are presented in Figure 3b. The adsorption quantity increases with contact time and the process can be divided into three steps. For DOX, a fast adsorption rate is revealed in the first 15 min, which is due to the large number of DOX molecules diffusing from the solution to the surface of the CDHA–MGO by a high mass-transfer driving force. Then, the adsorption rate gradually slows down between 15 and 60 min, and reaches equilibrium after 120 min. To gain more adsorption mechanisms of the CDHA–MGO for DOX, the adsorption data was analyzed by Lagergren’s pseudo-first-order kinetic model (Equation (3)) and Ho’s pseudo-second-order model (Equation (4)), and the linear fitting plots are shown in Figure S7a, b, respectively. The correlative parameters of the two models are revealed in Table 1. Compared with Lagergren’s pseudo-first-order model, Ho’s pseudo second-order model displays a higher correlation coefficient (R^2^ > 0.999). In addition, the calculated *Q_e_* value in Ho’s pseudo-second-order model approximates the experimental data *Q_e_*. Thus, the adsorption process of DOX agrees well with Ho’s pseudo-second-order kinetic model, clearly indicating a chemisorption process of CDHA–MGO for DOX. The probable driving forces in the adsorption process include strong hydrogen bonds, host–guest interaction, and hydrophobic interaction between the DOX and CDHA–MGO.

(3) $$\mathrm{In}(Q_{e}-Q_{t})={\mathrm{InQ}}_{e}-k_{1}t$$

(4) $$\frac{t}{Q_{t}}=\frac{1}{k_{2}Q_{e}^{2}}+\frac{t}{Q_{e}}$$

where *Q_e_* (mg·g^−1^) and *Q_t_* (mg·g^−1^) are the amounts of adsorbates adsorbed per unit mass of CDHA–MGO at equilibrium and *t* (min), respectively. *k*_1_ is the pseudo-first-order adsorption rate constant (min^−1^) and *k*_2_ is the pseudo-second-order adsorption rate constant (g (mg·min)^−1^).

To determine the saturated level of DOX loaded on CDHA–MGO, different amounts of DOX were added into the carrier solution of fixed concentration at pH 7.4. As shown in Figure 3c, it was found that the loading capacity of CDHA–MGO for DOX increases with the increasing initial DOX concentration, and it reaches 450 mg·g^−1^ when the initial DOX concentration is 0.5 mg/mL, which is still unsaturated. In order to obtain more insight into the adsorption behavior of CDHA–MGO for DOX, the Langmuir model (Equation (5)) and Freundlich model (Equation (6)) were used to assess the adsorption equilibrium data. It is well known that the Langmuir model is a theoretical model normally used for monolayer adsorption on a uniform surface, while the Freundlich model is suitable for multilayer adsorption in a heterogeneous system. The fitted plots from the two isotherms are shown in Figure S8, and Table 2 shows the relative parameters. By comparison of the linear correlation coefficients (R^2^), it was found that the Langmuir model is a better fit for the experimental results of the drug loading than the Freundlich model, indicating a monolayer adsorption. Through curve fitting of Langmuir isothermal adsorption, it was found that the maximum adsorption capacity of CDHA–MGO for DOX is 485.43 mg·g^−1^, as detailed in Table 2.

(5) $$\frac{1}{Q_{e}}=\frac{1}{Q_{m}}+\frac{1}{Q_{m}K_{L}}•\frac{1}{C_{e}}$$

(6) $${\mathrm{InQ}}_{e}={\mathrm{InK}}_{F}+\frac{1}{n}{\mathrm{InC}}_{e}$$

where *Q_e_* (mg/g) points to the amount of adsorbates at equilibrium. *C_e_* (mg/L) is the equilibrium concentration of adsorbate in the solution. *K_L_* (L/mg) and *K_F_* (L/mg) are the Langmuir and Freundlich constants, respectively, and *n* is the heterogeneity factor.

### 3.3. Photothermal Heating Effect of CDHA–MGO and In Vitro pH/NIR-Responsive DOX Release

**Figure 4:**
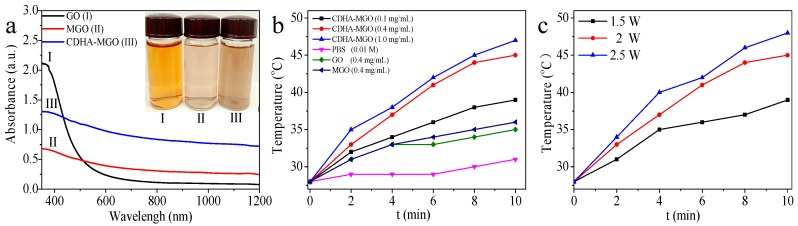
(**a**) UV-Vis-near-infrared (NIR) absorption spectrum and photograph (insert) of 0.25 mg/mL GO (I), MGO (II) and CDHA–MGO (III) solutions. (**b**) Photothermal heating curves of GO, MGO, and CDHA–MGO solution (PBS, pH 7.4) irradiated by NIR light at 2 W·cm^−2^. The concentrations of the GO and MGO solutions are 0.4 mg/mL, and the concentrations of the CDHA–MGO solutions are 0.1, 0.4, and 1 mg/mL; (**c**) photothermal heating curves of CDHA–MGO (0.4 mg/mL) at various NIR power intensities.

The UV-Vis-NIR spectrum illustrated in Figure 4a shows that GO, MGO, and CDHA–MGO exhibit NIR absorbances of 0.11, 0.30, and 0.83 at 808 nm, respectively. Compared with GO, the Fe_3_O_4_ nanoparticles deposition increased the optical absorption of MGO in the NIR region. For CDHA–MGO, the increased absorption may be attributed to the enhanced dispersity by further modification with CDHA [15,35]. As shown in Figure 4b, when irradiated by an 808 nm NIR laser at the power intensity of 2 W·cm^−2^, the solution temperature exceeded 45 °C within 10 min at the CDHA–MGO concentration of 0.4 mg/mL. In contrast, the temperature change of the pH 5.3 PBS solution (control) was only a slight increase (~5.0 °C) under the same NIR light irradiation condition. Compared with the temperature change of the GO (0.4 mg/mL) solution (~8.0 °C), the MGO (0.4 mg/mL) solution displayed a more obvious increase (~10.0 °C) according to the modification of Fe_3_O_4_. Furthermore, the photothermal heating effect of CDHA–MGO exhibited concentration-dependent and laser power intensity-dependent (from 1.5 to 2.5 W/cm^2^) behavior, as shown in Fibure 4b,c. The increase of CDHA–MGO concentration and the laser power exhibited remarkable enhancing photothermal conversion efficiency by NIR laser irradiation.

**Figure 5:**
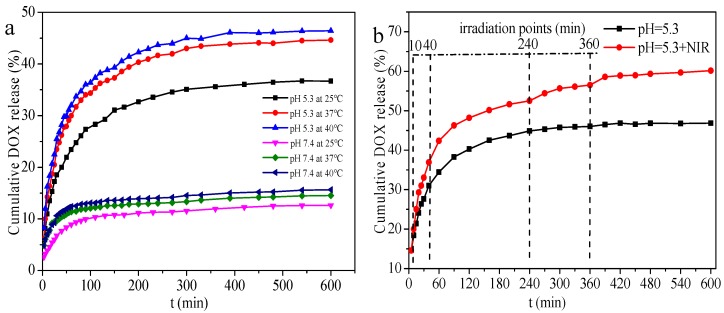
(**a**) Cumulative release of DOX from DOX-loaded CDHA–MGO (DOX@CDHA–MGO) at pH 7.4 and 5.3 (PBS buffer) at 25, 37, and 40 °C; (**b**) DOX release profiles of DOX@CDHA–MGO in PBS at pH 5.3 with periodic laser on/off irradiation (808 nm, 2 W·cm^−2^) at 37 °C.

To study the release behavior of DOX from DOX@CDHA–MGO, six sets of experiments with different pH (5.0 and 7.4) were performed at 25, 37, and 40 °C. The results are presented in Figure 5a. The DOX was released very slowly in the neutral system (pH 7.4), and only less than 15% of the loaded DOX was released after 10 h, even at 40 °C. However, in the weak acid system (pH 5.3), DOX was released quickly, and 35% of DOX was released in 8 h at 25 °C. Upon increasing the temperature up to 37 and 40 °C, about 45% and 47% of the total bound DOX was released in 8 h, respectively. The elevated released amount of DOX at low pH can be interpreted as being due to the reduction of the hydrogen bond interactions and host-guest interaction between the drugs and the CD, and also the decrease of electrostatic interaction between DOX and HA in the acidic microenvironments triggering more desired drug release [26]. The heat stimulation further dissociated these interactions between DOX and CDHA–MGO. Figure 5b compares the DOX-releasing kinetics from the CDHA–MGO dispersed in PBS solution of pH 5.3 at 37 °C, in the absence and presence of 5 min NIR light irradiation (808 nm, 2 W·cm^−2^) at certain releasing time points of 10, 40, 240, and 360 min. The cumulative release of DOX with NIR irradiation (60%) was about 1.3-fold greater than that without NIR irradiation (45%). The enhanced drug release under the NIR laser can be attributed to the photothermal effect of the CDHA–MGO. All the above results definitely illustrate that CDHA–MGO can realize pH and NIR dual-stimuli responsive drug release, confirming that CDHA–MGO could significantly increase the sensitivity of chemotherapy.

### 3.4. Intracellular DOX Release and In Vitro Cytotoxicity

**Figure 6:**
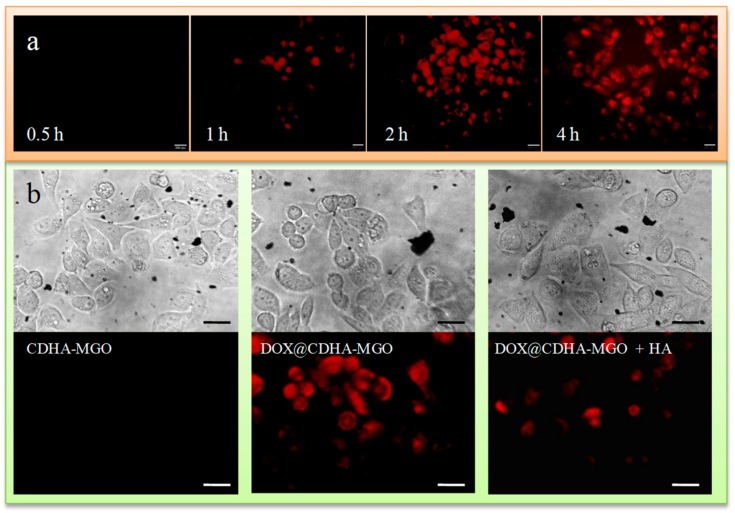
(**a**) Photomicrographs of BEL-7402 cells treated with DOX@CDHA–MGO (20 μg·mL^−1^) at 37 °C for incubation times of 0.5, 1, 2, and 4 h, observed under an inverted fluorescence microscope. (**b**) Fluorescence microscopy images of BEL-7402 cells incubated for 2 h at 37 °C with CDHA–MGO and DOX@CDHA–MGO in the presence or absence of free HA. Scale bar = 20 μm.

The anti-cancer activity of DOX is likely due to its intercalation into DNA, which may disrupt replication and transcription of genomic DNA and lead to the death of cancer cells. To understand intracellular uptake efficiency and the HA targeting ability, the DOX-loaded CDHA–MGO was evaluated using a fluorescence inverted microscope by incubating with BEL-7402 cells. As shown in Figure 6a, the obvious increasing red fluorescence of DOX could be clearly observed in the cytoplasm of BEL-7402 cells incubated with DOX@CDHA–MGO for 0.5 to 2 h, which implied that a large quantity of DOX released from DOX@CDHA–MGO had entered the tumor cells gradually and accumulated in cytoplasm. After 4 h of treatment, the fluorescence, especially in the nuclei, rose gradually. It can be concluded that DOX@CDHA–MGO is efficiently taken up by BEL-7402 cells accompanying abundant intracellular release, followed by the migration of DOX into the cell nucleus. In contrast, after incubation with DOX@CDHA–MGO and an excess amount of free HA for 2 h, weak red fluorescence was found in cytoplasm, as shown in Figure 6b. The weak fluorescence in cells was due to the fact that the CD44 receptor on cancer cells was occupied by free HA, resulting in a decrease of binding capacity between DOX@CDHA–MGO and CD44. This result clearly demonstrates that the DOX@CDHA–MGO is internalized into the cells via receptor-mediated endocytosis, based on the selective binding behavior between HA and CD44. Therefore, the HA-modified nanocomposites could be of potential for anti-cancer drug carriers.

**Figure 7:**
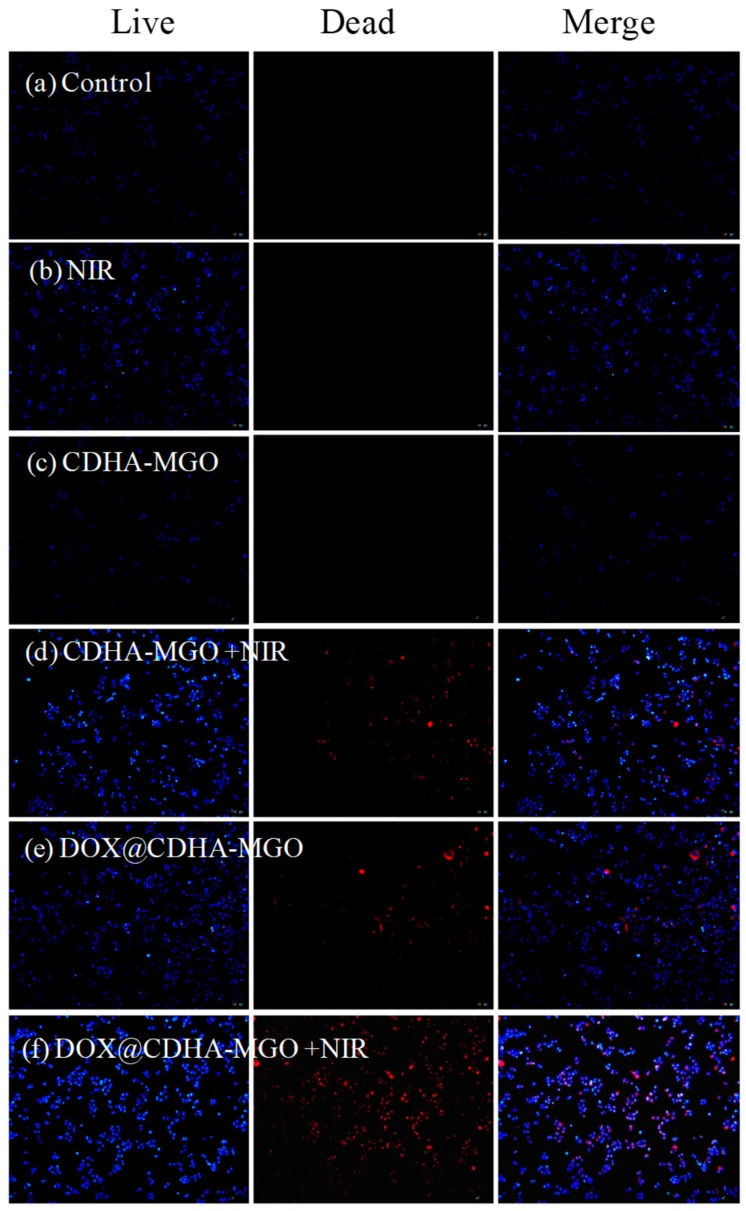
(**a**−**f**) Fluorescence microscopy images of BEL-7402 cells under different treatments via LIVE/DEAD staining at the concentration of 20 μg/mL, with or without NIR laser irradiation (808 nm, 2 W·cm^−2^) at 37 °C. Blue: Live cells. Red: Dead cells. Bar = 50 μm.

In order to evaluate the therapeutic effects of CDHA–MGO and DOX@CDHA–MGO, the cell viability with photothermal and chemo-photothermal treatments was measured. As shown in Figure 7, the qualitative observations with fluorescence microscopy images were obtained by LIVE/DEAD co-staining the BEL-7402 cells, which clearly showed that DOX@CDHA–MGO with NIR irradiation mediated the highest rate of cell death compared to a single treatment of DOX@CDHA–MGO (chemotherapy) or CDHA–MGO with NIR irradiation (photothermal therapy). Obviously, the results illustrated that the CDHA–MGO nanocomposite could transform laser energy into heat energy, which could induce the drug releasing validly, and reduce adverse side effects in normal tissues as a photothermal agent.

**Figure 8:**
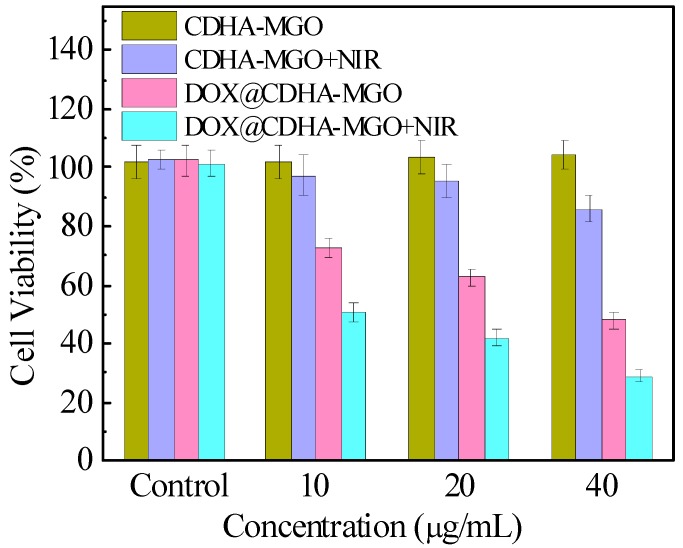
In vitro cytotoxicity of CDHA–MGO and DOX@CDHA–MGO in the presence or absence of NIR irradiation at 2 W·cm^−2^ for 5 min, as measured in terms of cell viability.

To further verify the cytotoxicity in vitro, the cell viability was determined by standard MTT assay. As depicted in Figure 8, CDHA–MGO had negligible cytotoxicity against BEL-7402 cells, even at high concentrations. However, CDHA–MGO exhibited some cytotoxic effects in cells under NIR irradiation for 5 min, suggesting that they could serve as potential photothermal therapeutic agent in treating cancer cells. The dose-dependent cell viability for DOX@CDHA–MGO significantly reduced with increasing concentration. Furthermore, at each concentration, the cytotoxicity of DOX@CDHA–MGO was remarkably lower than that of DOX@CDHA–MGO irradiated by NIR for 5 min, likely due to the photothermal release behaviors of the carrier. These fascinating properties give a great opportunity for CDHA–MGO nanocarriers to be potential candidates for targeted delivery and controlled release of DOX for cancer treatment.

## 4. Conclusions

In summary, a β-CDHA polymer-modified magnetic graphene oxide nanocomposite (CDHA–MGO), as a novel multifunctional drug delivery system, was successfully constructed for synergistic chemo-photothermal therapy. CDHA–MGO possesses the following important features: (1) High loading efficiency of antitumor drug DOX for chemotherapy, (2) easy separation and magnetic targeting properties with an external magnetic field, (3) high NIR absorption and efficient heat transformation for photothermal therapy, (4) CD44-targeting for accumulation within tumor cells via HA conjugation, and (5) controlled release characteristics including NIR/heat-stimulation and pH-responsive. Such beneficial functions concentrated in one drug delivery platform could effectively improve the therapeutic effect for against cancer. Hence, we believe that the presented CDHA–MGO has promising potential for efficient anti-cancer therapy and other biomedical applications.

## Figures and Tables

**Table 1:** Kinetic parameters for the loading of DOX by CDHA–MGO.

Lagergren’s Pseudo-first-order Kinetic Model			Ho’s Pseudo-second-order Kinetic Model		
*Q_e_* (mg/g)	*k*_1_ (h^−1^)	R^2^	*Q_e_* (mg/g)	*k*_2_ (g (mg h)^−1^)	R^2^
3.748	0.009210	0.7516	79.05	0.04245	0.9999

**Table 2:** The adsorption isotherm parameters for the loading of DOX by CDHA–MGO.

Langmuir Isotherm Model			Freundlich Isotherm Model		
*Q_m_* (mg/g)	*K_L_* (L/mg)	R^2^	*n*	*K_F_* (L/mg)	R^2^
485.4	30.77	0.9863	3.890	1.293	0.9742

## Supplementary Materials

The following are available online at http://www.mdpi.com/2073-4360/11/1/133/s1. Figure S1. ^1^H NMR and ^13^C NMR spectra of mono-6-deoxyl-6-ethylenediamino-β-CD; Figure S2. ^1^H NMR spectra of CDHA; Figure S3. Hydrodynamic diameter distributions of MGO and CDHA–MGO; Figure S4. N_2_ adsorption–desorption isotherm curve of MGO and CDHA–MGO; Figure S5. Plot of calibration curves for DOX solution with different concentrations; Figure S6. Influence of pH on the adsorption of DOX onto CDHA–MGO; Figure S7. Linear fitting plots of Lagergren’s pseudo-first-order and Ho’s pseudo-second-order equations; Figure S8. Langmuir plot and Freundlich plot for adsorption of DOX.
