# Supplementary material for: β-Cyclodextrin–Hyaluronic Acid Polymer Functionalized Magnetic Graphene Oxide Nanocomposites for Targeted Photo-Chemotherapy of Tumor Cells
Source: Polymers (Basel). 2019 Jan 14;11(1):133. doi: 10.3390/polym11010133 (PMC6401939; doi:10.3390/polym11010133)
Supplement: Supplementary file 1 [file polymers-11-00133-s001.pdf]

Supplementary Material

β-Cyclodextrin-Hyaluronic Acid Polymer Functionalized Magnetic Graphene Oxide Nanocomposites for Targeted Photo-Chemotherapy of Tumor Cells

Wenting Liang [1,*], Yu Huang [1], Dongtao Lu [1], Xuewen Ma [1], Tao Gong [2], Xiaodong Cui [1], Baofeng Yu [2,*], Cheng Yang [3,*], Chuan Dong [1] and Shaomin Shuang [1,*]

Fig.S1. **^{1}H NMR and ^{13}C NMR spectra of mono-6-deoxyl-6-ethylenediamino-β-CD.**

Fig.S2. **^{1}H NMR spectra of CD-HA.**

Fig.S3. Hydrodynamic diameter distributions of MGO and CDHA-MGO.

Fig.S4. **N_2 adsorption-desorption isotherm curve of MGO and CDHA-MGO.**

Fig.S5. Plot of calibration curves for DOX solution with different concentration.

Fig.S6. Influence of pH on the adsorption of DOX onto CDHA-MGO.

Fig.S7. Linear fitting plots of Lagergren's pseudo-first-order and Ho's pseudo-second-order equations.

Fig.S8. Langmuir plot and Freundlich plot for adsorption of DOX.

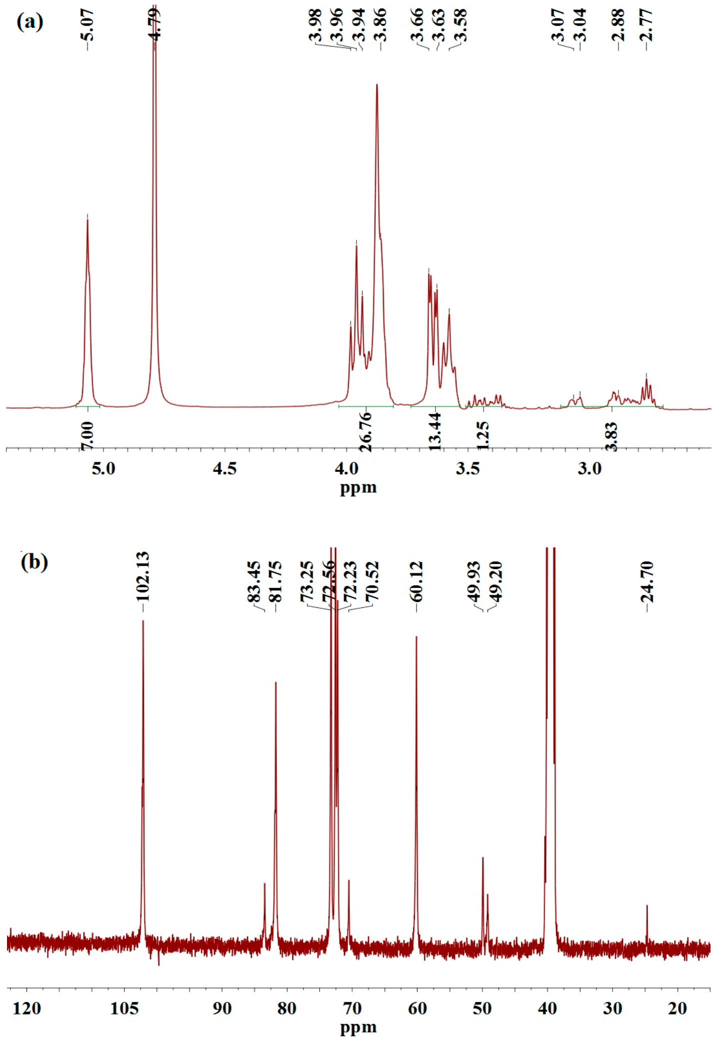

Fig. S1. (a) ^{1}H NMR spectra of mono-6-deoxyl-6-ethylenediamino-β-CD (400 MHz, D_2O, TMS): δ=5.07 (s, 7 H, H of C-1 of β-CD), 3.55-3.98 (m, 40 H, H of C-3, C-5, C-6, C-2, C-4 of β-CD), 2.86-3.04 (m, 4 H, -NH-CH_2CH_2-NH-); (b) ^{13}C NMR spectra of mono-6-deoxyl-6-ethylenediamino-β-CD (101 MHz, DMSO): δ=102.13, 83.45, 81.75, 74.80, 73.63, 72.90, 72.23 , 70.52, 60.12, 49.93, 49.20, 24.70 ppm

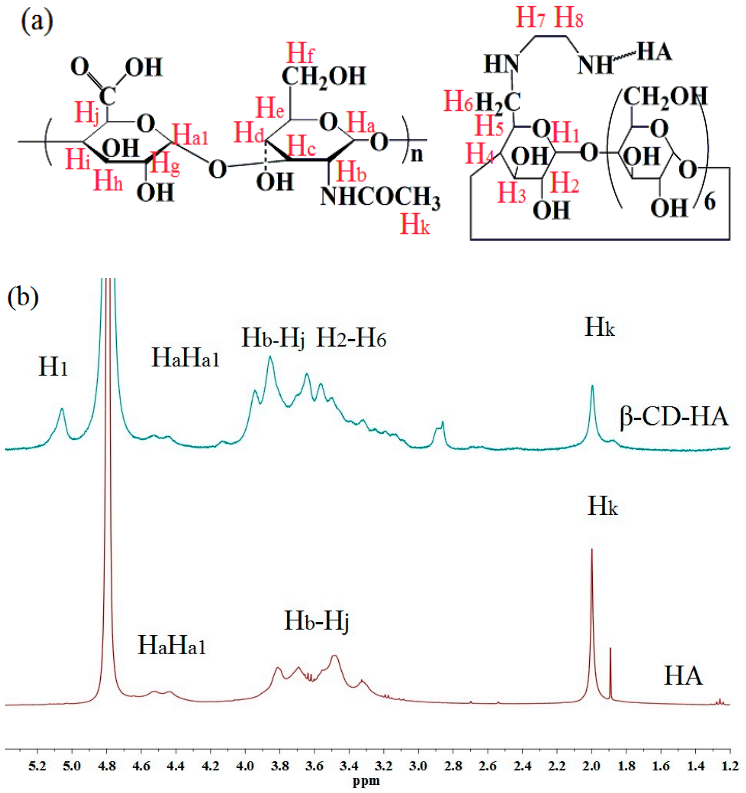

Fig. S2. ^{1}H NMR spectra of CD-HA (600 MHz, D_2O, ppm):δ=1.99 (s, 3 H, H of CH_3 group of HA), 3.02-3.94(m, 16.44 H, H of HA and C-3, C-5, C-6, C-2, C-4 and methylene on ethane diamine group of β-CD), 4.47-4.52(m, 2 H, H of HA), 5.02-5.06 (m, 2.25 H, H of C-1 β-CD).

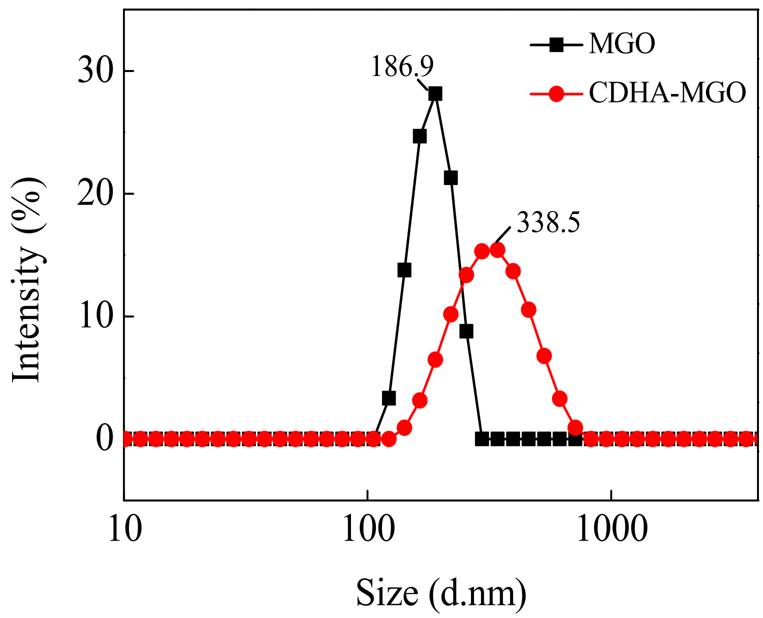

Fig.S3. Hydrodynamic diameter distributions of MGO and CDHA-MGO.

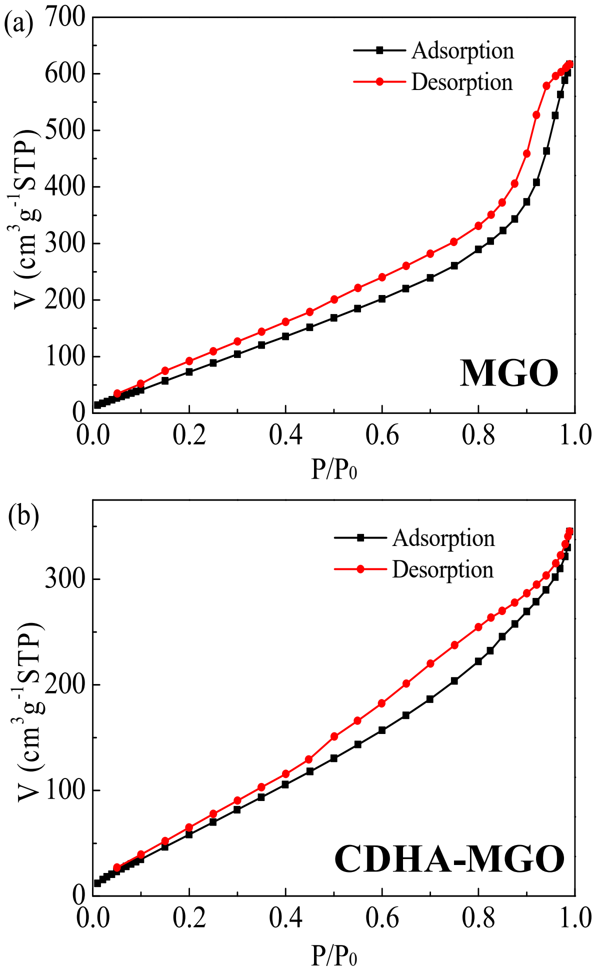

Fig. S4. N_2 adsorption-desorption isotherm curve of MGO (a) and CDHA-MGO (b).

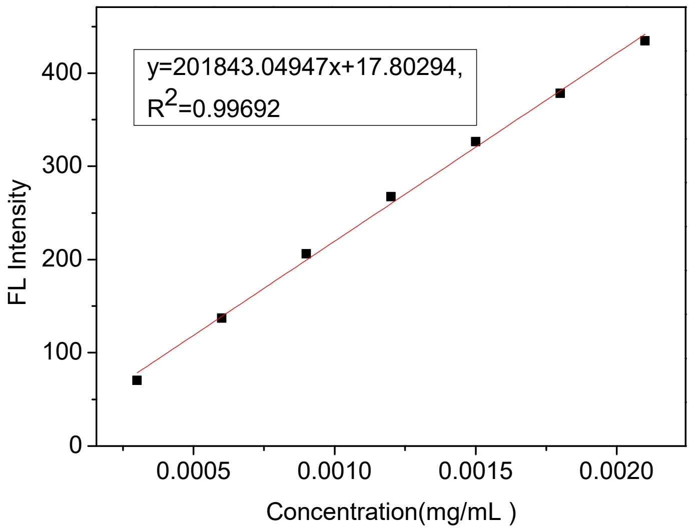

Fig. S5. Plot of calibration curves for DOX solution with different concentration.

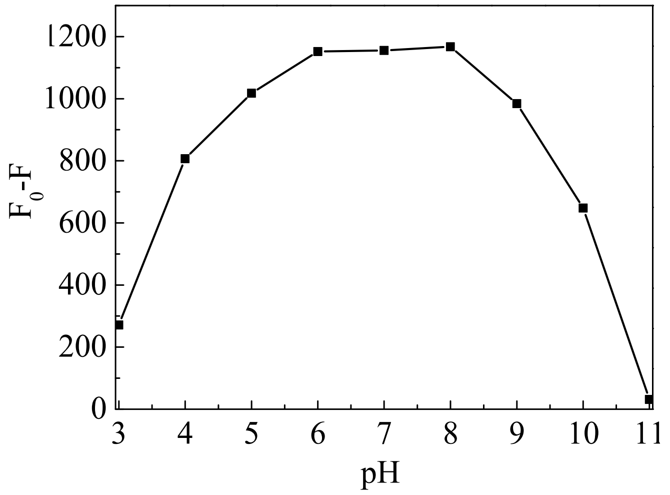

Fig. S6. Influence of pH on the adsorption of DOX onto CDHA-MGO.

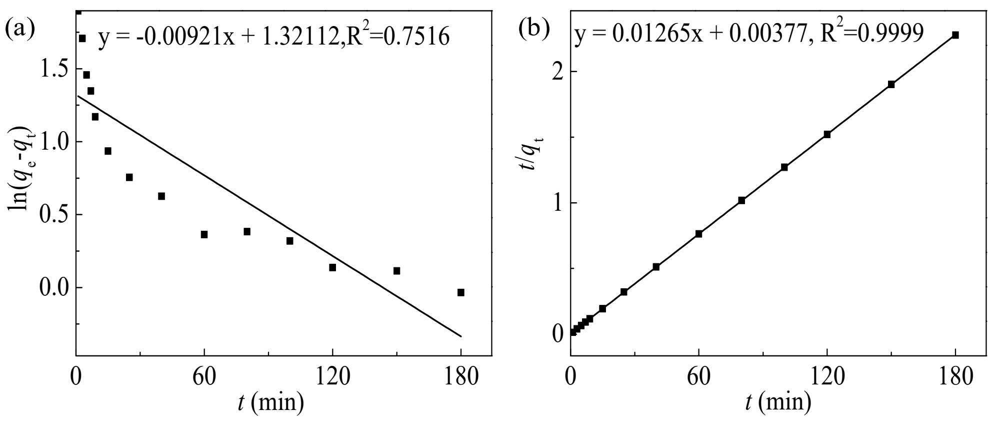

Fig. S7. The linear fitting plots of Lagergren's pseudo-first-order (a) and Ho's pseudo-second-order (b) equations.

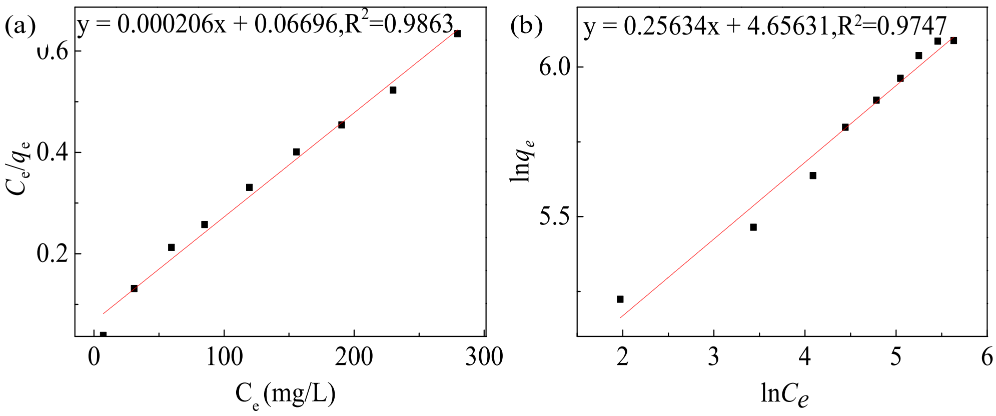

Fig. S8. Langmuir plot illustrating the linear dependences of $1/Q_e$ on $1/C_e$ (a), and Freundlich plot illustrating the linear dependences of $\ln Q_e$ on $\ln C_e$ (b) for adsorption of DOX.
